# Two New *Kremastochrysopsis* species, *K. austriaca* sp. nov. and *K. americana* sp. nov. (Chrysophyceae)^1^

**DOI:** 10.1111/jpy.12937

**Published:** 2019-11-27

**Authors:** Daniel Remias, Lenka Procházková, Linda Nedbalová, Robert A. Andersen, K. Valentin

**Affiliations:** ^1^ School of Engineering University of Applied Sciences Upper Austria 4600 Wels Austria; ^2^ Department of Ecology Faculty of Science Charles University 12844 Prague Czech Republic; ^3^ Friday Harbor Laboratories University of Washington Friday Harbor Washington DC 98250 USA

**Keywords:** *Chromophyton*, *Kremastochrysis*, psychrophilic, snow algae, substitutional saturation

## Abstract

Melting summer snow in the Austrian Alps exhibited a yellowish bloom that was mainly comprised of an unidentified unicellular chrysophyte. Molecular data (18S rRNA and *rbc*L genes) showed a close relationship to published sequences from an American pond alga formerly identified as *Kremastochrysis* sp. The genera *Kremastochrysis* and *Kremastochrysopsis* are morphologically distinguished by the number of flagella observed with the light microscope, and therefore we assigned the Austrian snow alga and an American pond alga to the genus *Kremastochrysopsis*. Transmission and scanning electron microscopy revealed that swimming cells had two flagella oriented in opposite directions, typical for the Hibberdiales. Molecular phylogenetic analyses showed that both new species were closely related to *Hibberdia. Kremastochrysopsis ocellata*, the type species and only known species, has two chloroplasts per cell and the zoospores have red eyespots. Our two organisms had only a single chloroplast and no zoospore eyespot, but their gene sequences differed substantially. Therefore, we described two new species, *Kremastochrysopsis austriaca* sp. nov and *Kremstochrysopsis americana* sp. nov. When grown in culture, both taxa showed a characteristic hyponeustonic growth (hanging below the water surface), whereas older immotile cells grew at the bottom of the culture vessel. Ecologically, *Kremastochrysopsis austriaca* sp. nov., which caused snow discolorations, had no close phylogenetic relationships to other psychrophilic chrysophytes, for example, *Chromulina chionophilia*,* Hydrurus* sp., and *Ochromonas‐*like flagellates.

The Chrysophyceae is a diverse class of heterokont (stramenopile) microorganisms that occur in a variety of freshwater and marine habitats (Pascher 1912, 1913, Bourrelly 1957, Starmach 1985, Nicholls and Wujek 2015). Several chrysophytes (e.g., *Chromophyton, Chrysotilos, Kremastochrysis*, and *Kremastochrysopsis*) are associated with the air–water interface, or neuston. Of these, *Kremastochrysis* and *Kremastochrysopsis* hang down from the air–water interface and are hyponeustonic organisms (Pascher 1942). Conversely, the more commonly occurring *Chromophyton* grows up from the air–water interface and is an epineustonic organism (Woronin 1880, Lund 1942, Vischer 1943, Petersen and Hansen 1958). *Kremastochrysis* has been rarely reported since its description by Pascher (1942). The type species, *Kremastochrysis pendens*, has a single chloroplast, and its zoospores have two flagella but no eyespot was reported. Pascher (1942) described a second species, *Kremastochrysis ocellata*, that has two chloroplasts, and its zoospores are uniflagellate and possess an eyespot on one of the plastids. Bourrelly (1957) proposed the genus *Kremastochrysopsis* (*Kremastochrysopsis ocellata*) because of its single visible flagellum when viewed with a light microscope. Thus, *Kremastochrysis* and *Kremastochrysopsis* differ by number of flagella visible by light microscopy. Finally, a third species, *Kremastochrysis minor* Catalan has been described (Catalan 1987).

The occurrence of one or two flagella (and their relative lengths) has long been an important character for classifying taxa in the Chrysophyceae (e.g., Pascher 1913; Chromulinales, Isochrysidales, and Ochromonadales). This character reached a pinnacle when Bourrelly (1965) established subclasses within the Chrysophyceae based on flagellar patterns (see also Bourrelly 1981, Starmach 1985, Andersen 2007). Electron microscopy showed that a second, very short flagellum was present on organisms previously believed to be uniflagellates (e.g., Rouiller and Fauré‐Fremiet 1958, Belcher and Swale 1967, 1971, Hibberd 1976, Andersen 1986, 1989). However, despite the discovery of a second flagellum for “uniflagellate” organisms, classification based upon flagellar number has persisted and is supported by molecular evidence. One early molecular phylogenetic study suggested that uniflagellate and biflagellate lineages may be monophyletic, supporting the Pascher/Bourrelly classifications (Andersen et al. 1999). However, more recent molecular phylogenetic studies recovered only smaller clades that were strictly uniflagellate or biflagellate, while a few clades were recovered with both flagellar types (e.g., Andersen 2007, Grossmann et al. 2016, Andersen et al. 2017, Pusztai and Škaloud 2019). Today, flagellar number (as viewed in the light microscope) carries phylogenetic signal for smaller clades, but these clades characterize orders, families or genera, not subclasses.

This paper addresses the molecular phylogenetic relationships for two new species of *Kremastochrysopsis*. One strain was previously identified as *Kremastochrysis* sp. (Andersen 2007) and the other strain was recently isolated from melting alpine snow, that is a non‐hyponeustonic habitat. We discuss the relationships of *Kremastochrysis, Kremastochrysopsis* and related chrysophytes, including comments about snow habitats.

## Materials and Methods

### Collection, culturing, and microscopy

Strain DR75b was collected from melting snow at an alpine meadow May 5, 2017 in Austria, province Tyrol, district Imst, east of Kühtai village. The GPS was 47°13.223 N, 11°02.431 E at an elevation of 1998 m. A unialgal strain was isolated from petri dishes with solidified DY‐Vm medium (NaH_2_PO_4_·H_2_O substituted for Na_2_ β‐glycerophosphate; https://ncma.bigelow.org/media/wysiwyg/Algal_recipes/NCMA_algal_medium_DY-V.pdf) and 1.6% agar, kept at 5°C. Strain CCMP 260 was collected from a pond in Massachusetts, USA, by Ralph Lewin, but other information is missing (e.g., date, precise location). For morphological studies, both strains were grown at room temperature (varying between 10°C and 25°C) in DY‐V medium (with Na_2_ β‐glycerophosphate) or in a biphasic soil–water medium. Cells were observed with a Leica DM RB light microscope (Leica Microsystems Inc., Buffalo Grove, IL, USA) equipped with differential interference contrast, phase contrast, brightfield, and darkfield optics; cells were photographed using a Canon T6i DSLR camera (Canon USA Inc., Melville, NY, USA).

For transmission electron microscopy, strain DR75b was grown at 1°C and fixed as described previously (Procházková et al. 2018). TEM grids were examined with a JEOL 1011 TEM (JEOL Ltd., Tokyo, Japan) at 80 kV. Photomicrographs were taken with a Veleta CCD camera and iTEM 5.1 software (Olympus Soft Imaging Solution GmbH, Münster, Germany). For scanning electron microscopy, strain DR75b was grown at 5°C and strain CCMP260 was grown at 18°C; both strains were fixed as described in Hanousková et al. (2019). SEM gold‐coated coverslips were observed at 80V with a JEOL 6380 LV (JEOL Ltd.).

### Molecular phylogeny

Total genomic DNA was extracted from strain DR75b as described in Procházková et al. (2018). The 18S rRNA and *rbc*L gene regions were amplified from DNA isolates by PCR using existing primers. For the 18S rRNA gene sequence, there were two PCR reactions: the forward primer SSU1F (CCT GGT TGA TCC TGC CAG T; Medlin et al. 1988) with the reverse primer SSU1295R (TCA GCC TTG CGA CCA TAC) and the forward primer SSU1065F (TCA GAG GTG AAA TTC TTG GAT T) with the reverse primer SSU1954R (CCT TGT TAC GAC TTC TCC TTC C; Yang et al. 2012). For the *rbc*L sequence, there was one reaction using the forward primer rbcL46F (CGT TAY GAA TCT GGT GTA ATH CC) and the reverse primer rbcL1425R (GTA TCT GTT GAW GWA TAG TCR AA; Andersen et al. 2017). Amplification and sequencing reactions for these markers were identical to those described by Procházková et al. (2018). The newly generated sequences of the strain DR75b are available under GenBank accession numbers: MK614366 – 18S rRNA gene; MK614367 – *rbc*L. The sequences of the second investigated strain CCMP260 were published in previous studies (Andersen et al. 1999, Andersen 2007).

Two different alignments were constructed for the phylogenetic analyses, based on the 18S rRNA and *rbc*L gene sequences. The sequences were selected according to the publications of Kristiansen and Škaloud (2017) and Andersen (2007) to encompass all chrysophycean lineages. The 18S rRNA gene alignment contained 88 sequences (1581 bp); the initial *rbc*L matrix consisted of 49 sequences (921 bp). Second, to remove saturated nucleotide sites of the third *rbc*L codon partition, a modified site‐stripping approach was applied (Waddell et al. 1999, Škaloud et al. 2013). Site‐specific rates were calculated with the “Substitution Rates” standard analysis implemented in HyPhy (Pond et al. 2005), under a global GTR+G+I model using the inferred Maximum Likelihood phylogeny as a guide tree. 66% of fast‐evolving sites in the third codon position of *rbc*L were removed using SiteStripper (Verbruggen 2012), according to the rates file generated in HyPhy. The stripped *rbc*L alignment was 816 bp long. Third, an *rbc*L matrix consisting of first and second *rbc*L codon positions only was prepared (alignment was 614 bp long). The outgroup taxa (*Synchroma* and *Nannochloropsis*) were selected according to the results of the recent multigene phylogenetic analysis of stramenopiles by Yang et al. (2012). The best‐fit nucleotide substitution model was estimated by jModeltest 2.0.1 (Posada 2008). Based on the Akaike Information Criterion, the ‘GTR+I+G’ model was selected for 18S rRNA gene. Three partitions were set for *rbc*L gene sequences and GTR+I+G substitution model was applied for each of three codon positions. The 18S rRNA gene and *rbc*L phylogenetic trees were inferred by Bayesian Inference and Maximum Likelihood according to Nedbalová et al. (2017), with the minor modification that Markov Chain Monte Carlo runs were carried out for three million generations in Bayesian Interference. Convergence of the two cold chains was checked by the average standard deviation of split frequencies (0.001228 and 0.001004 for 18S rRNA gene and *rbc*L data set, respectively). Bootstrap analyses and Bayesian posterior probabilities were performed as described by Nedbalová et al. (2017).

## Results

**Figure 1:**
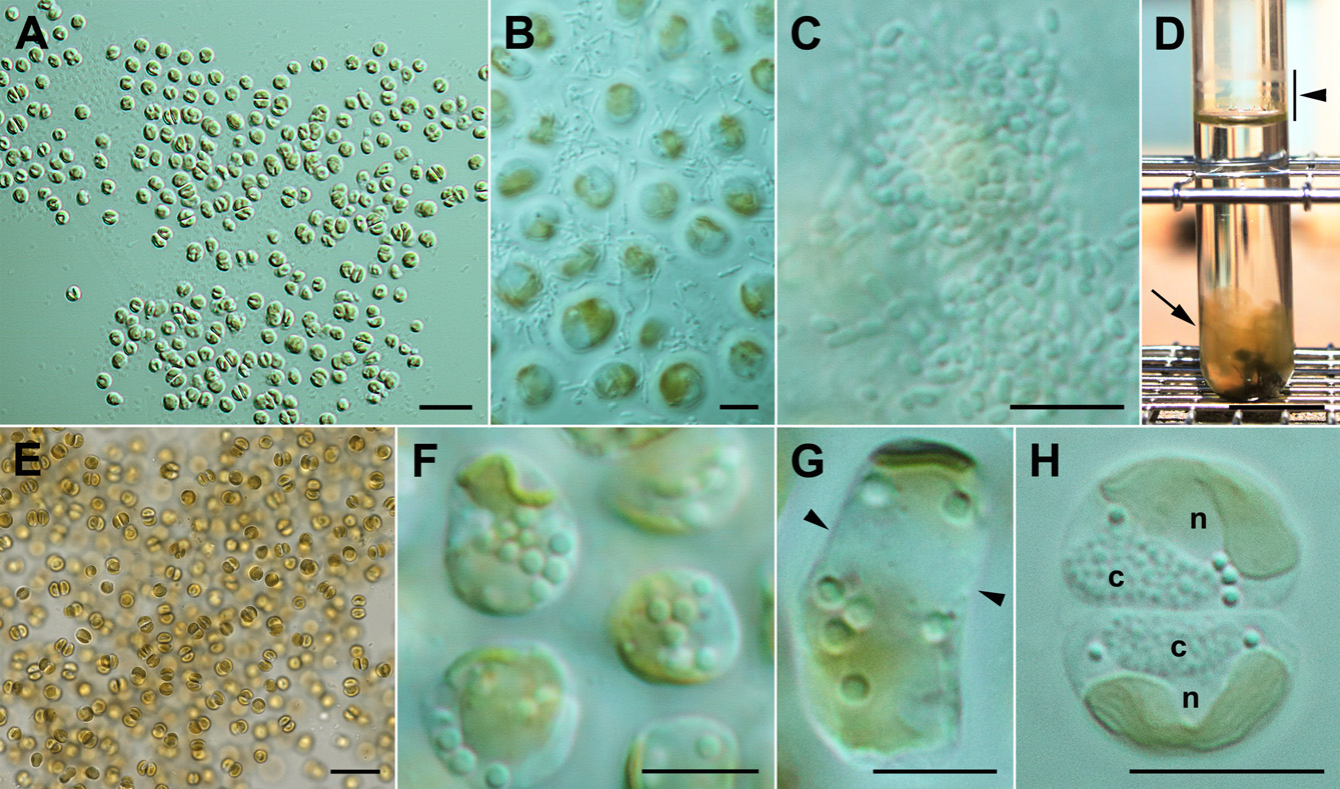
*Kremastochrysopsis austriaca* sp. nov. (A) Monolayer of cells below the air–water interface. Scale bar = 20 μm. (B) Cells just below the air–water interface surrounded by bacteria. Scale bar = 5 μm. (C) Bacterial plaques above the cells exactly at the air–water interface. Scale bar = 5 μm. (D) Test tube (20 mm diameter) showing dried cells above the air–water interface (bar, arrowhead) caused by evaporation from the test tube and a cloud of palmelloid cells near the bottom of the tube (arrow). Biphasic soil–water medium. Scale bar = 20 mm. (E) Palmelloid cell mass from near the bottom of a test tube, likely rich in carotenoids. DY‐V medium. Scale bar = 20 μm. (F) Non‐motile cells showing the parietal chloroplast and lipid droplets. Scale bar = 5 μm. (G) A rectangular dividing cell showing the future division plane (arrowheads). Scale bar = 5 μm. (H) Two daughter cells showing typical hemispherical shapes. Note the granular chrysolaminarin vacuoles (c) and the nuclei (n). Scale bar = 5 μm. [Color figure can be viewed at http://www.wileyonlinelibrary.com]

**Figure 2:**
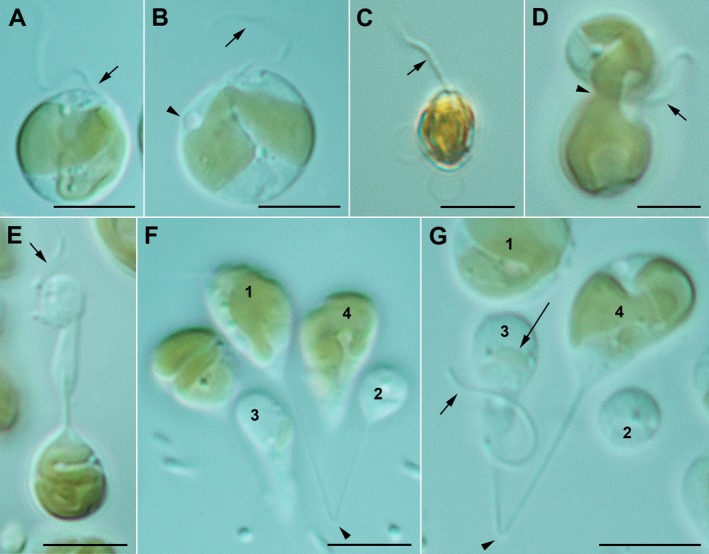
*Kremastochrysopsis austriaca* sp. nov. Scale bars = 5 μm. (A) Flagellate cell showing the single visible flagellum (arrow). (B) Flagellate cell showing the flagellum (arrow) and contractile vacuole (arrowhead). Note the narrow bridge connecting the two chloroplast lobes. (C) Flagellate cell stained with Lugol's solution showing a single flagellum (arrow). Note that there is no evidence of a second flagellum. (D) A dividing flagellate cell. Note that one future daughter cell has a flagellum but the other does not. Note the chloroplast lobe pinching at the plane where the daughter cells are pinching apart. (E) An odd dividing flagellate cell where one future daughter cell has a flagellum (arrow) but lacks a chloroplast. (F) Image showing a long thin cytoplasmic strand (arrowhead) that connects cells 1 and 2. Note that cell 2 is smaller and lacks a chloroplast. Cell 1 had a flagellum but it is out of the plane of view. (G) Image similar to F but focused slightly different to show the cytoplasmic strain connecting cells 3 and 4. Note the flagellum on the smaller cell (short arrow) and the very small chloroplast in cell 3 (long arrow). [Color figure can be viewed at http://www.wileyonlinelibrary.com]

**Figure 3:**
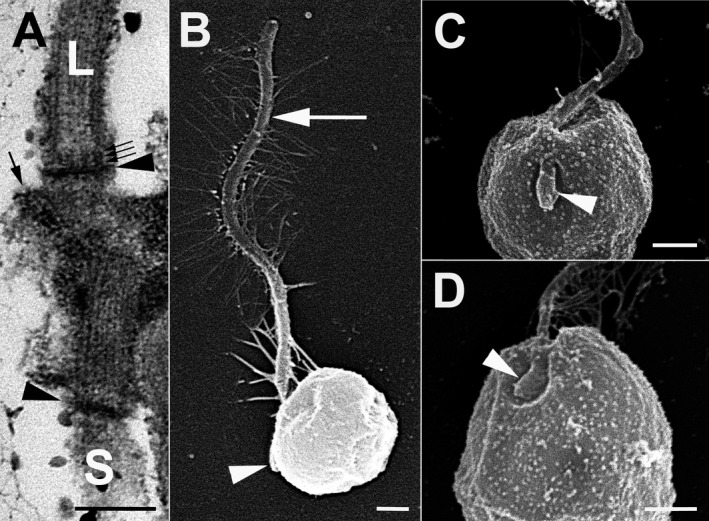
*Kremastochrysopsis austriaca* sp. nov. and *K. americana* sp. nov. (A) *K. austriaca*. TEM image of the long flagellum (L), short flagellum (S), and their basal bodies. Note the dense transitional plates (arrowheads), the three‐gyre transitional helix (small arrows), and the microtubules of root R_1_ (large arrow). Scale bar = 200 nm. (B) *K. austriaca*. SEM image showing the long flagellum (arrow) and hidden short flagellum (arrowhead). Note that the cell shrank during dehydration. Scale bar = 400 nm. (C) *K. austriaca*. SEM image showing the short flagellum (arrowhead) lying in a shallow depression. Scale bar = 500 nm. (D) *K. americana*. SEM image showing the short flagellum (arrowhead) lying in a shallow depression. Scale bar = 500 nm.

***Description. Kremastochrysopsis austriaca*** **Remias, Procházková & R. A. Andersen sp. nov.** (Figs. 1, 2, 3)

*Diagnosis:* Non‐motile vegetative cells without cell walls, ~(4)5–8(10) μm diameter; cells with one chloroplast, up to three contractile vacuoles, lipid droplets, and chrysolaminarin vacuole; both non‐motile and motile cells capable of cell division; zoospores formed directly from vegetative cells; zoospores generally oval in shape, 4–6 × 6–8 μm; zoospores uniflagellate, with one plastid and no eyespot; cysts not observed; 18S rRNA and *rbc*L gene sequences distinctive.

*Holotype here designated:* NY 02666691; a permanent microscope slide prepared from culture strain DR75b and deposited in the New York Botanical Garden herbarium, New York City, NY USA.

*Isotype here designated:* NY 02666692; a permanent microscope slide prepared from culture strain DR75b and deposited in the New York Botanical Garden herbarium, New York City, NY USA.

*Isotype here designated:* WU 0106471; cells embedded in a resin block from strain DR75b and deposited in the herbarium of the University of Vienna, Austria.

*Type locality:* Kühtai, district Imst, Tyrol, Austria, Europe; melting winter snow above an alpine meadow (47°13.223′ N, 11°02.431′ E).

*Etymology:* the specific epithet “austriaca” refers to Austria, the country where the alga was collected.

**Figure 4:**
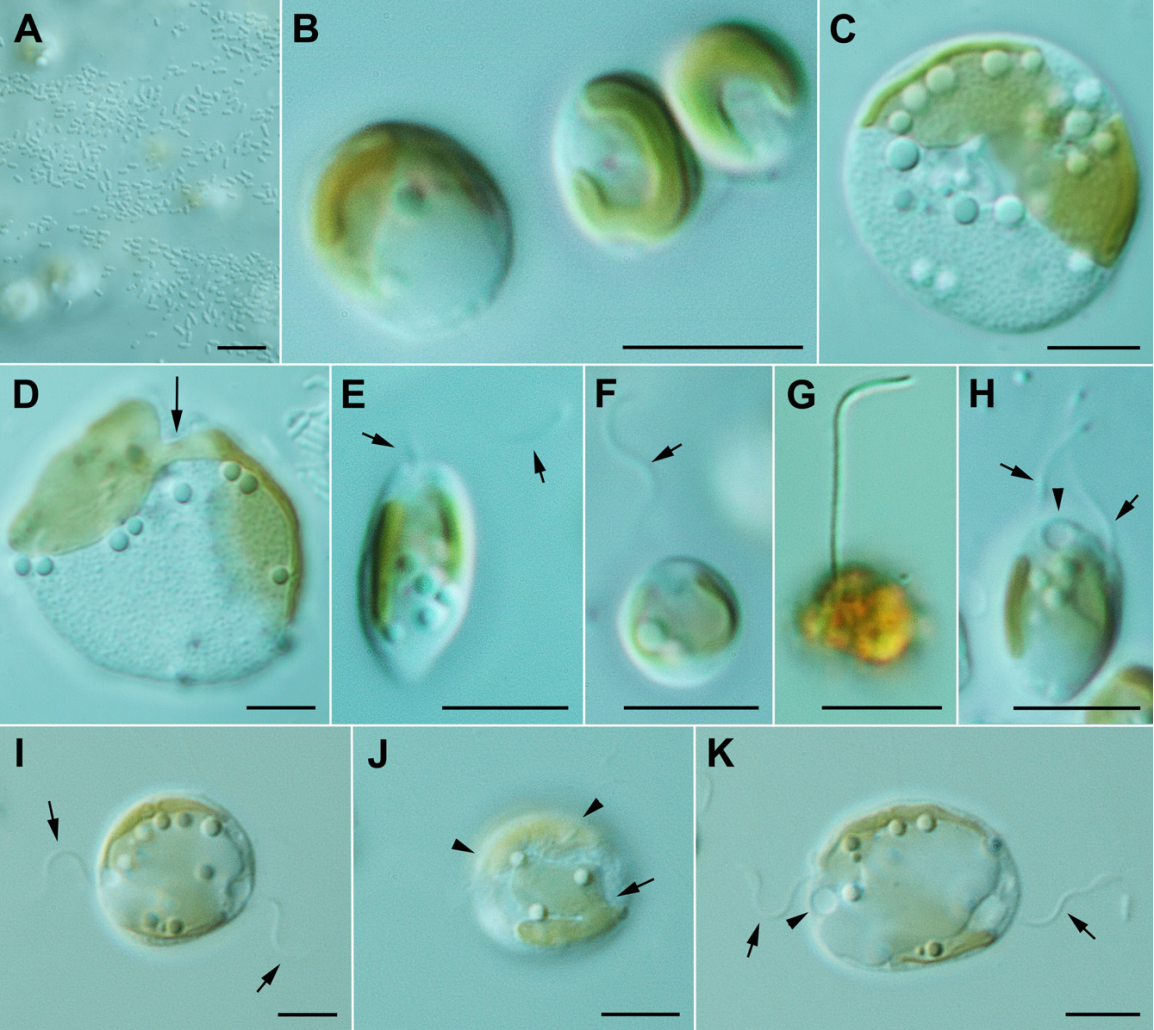
*Kremastochrysopsis americana* sp. nov. Scale bars = 5 μm. (A) Image of the air–water interface showing the bacterial plaques and algal cells out of focus below the interface. (B) Three vegetative cells showing the parietal chloroplast. (C) A large and very flattened cell showing the bilobed chloroplast, numerous lipid droplets and a granular chrysolaminarin vacuole. (D) A large, flattened cell showing the bridge (arrow) between the two chloroplast lobes. (E) An elongate swimming cell with a single flagellum (arrows). (F) A spherical swimming cell with a single flagellum (arrow). (G) A swimming cell stained with Lugol's solution showing the single flagellum but no evidence of a second flagellum. (H) A dividing swimming cell showing the two visible flagella (arrows) and a contractile vacuole (arrowhead). (I–K) Three images of the same dividing flagellate cell: (I) The cell has flagella at the opposite poles of the cell (arrows). (J) A different focal plane showing that the chloroplast had already divided. Note the upper plastid (arrowheads) and the lower plastid with a deep division between the chloroplast lobes (arrow). (K) The same cell several minutes later that was more elongated. Note the two flagella (arrows) and the contractile vacuole (arrowhead). [Color figure can be viewed at http://www.wileyonlinelibrary.com]

***Kremastochrysopsis americana*** **R. A. Andersen, Procházková & Remias sp. nov.** (Figs. 3, 4)

*Diagnosis:* Non‐motile vegetative cells without cell walls, ~(4)5–10(12) μm in diameter; cells with one chloroplast, 1–2 contractile vacuoles; lipid droplets, and chrysolaminarin vacuole; both non‐motile and motile cells capable of cell division; zoospores were oval in shape, 5–6 × 6–10 μm, uniflagellate when viewed in the light microscope, with one plastid and no eyespot, 1–2 contractile vacuoles, chrysolaminarin vacuole and fat droplets; cysts not observed; 18S rRNA and *rbc*L gene sequences distinctive.

*Holotype here designated:* NY 02666693; a permanent microscope slide prepared from culture strain CCMP260 and deposited in the New York Botanical Garden herbarium, New York City, NY USA.

*Isotype here designated:* NY 02666694; a permanent microscope slide prepared from culture strain CCMP260 and deposited in the New York Botanical Garden herbarium, New York City, NY USA.

*Type locality:* unknown pond, Massachusetts (presumably near Woods Hole), USA.

*Etymology:* the epithet “americana” refers to the continent where the alga was collected.

***Kremastochrysis pendens*** **Pascher lectotype specimen designated here: figure 9** in Pascher, A. 1942. *Beihefte zum Botanischen Centralblatt. Abteilung A. Morphologie und Physiologie der Pflanzen* 61:467.

***Kremastochrysopsis ocellata*** **(Pascher) Bourrelly lectotype specimen designated here: figure 12c** in Pascher, A. 1942. *Beihefte zum Botanischen Centralblatt. Abteilung A. Morphologie und Physiologie der Pflanzen* 61:470.

*Kremastochrysopsis austriaca* sp. nov. strain DR75b grew just below the air–water interface (hyponeustonically) when grown in DY‐V or soil–water medium. These cells formed a monolayer that was composed of immobile and swimming cells (Fig. 1A). Bacteria formed a covering around and over the cells (Fig. 1, B and C). In older cultures, cells grew at the air–water interface and at the bottom of the culture tube (Fig. 1D). Cells at the bottom of test tubes formed palmelloid “clouds” of cells held together by a colonial gel (Fig. 1E). The gel margin stained with brilliant cresyl blue (not shown). Immobile cells were (4)5–8(10) μm in size, lacked a cell wall, had a single parietal chloroplast, 1–2 contractile vacuoles, a chrysolaminarin vacuole, and several lipid droplets (Fig. 1, F–H). The chloroplast was sometimes deeply lobed, but always connected by a bridge between the two lobes. Immobile cells divided by first elongating and dividing the chloroplast into two plastids (Fig. 1G) and then by forming hemispherical daughter cells after cytokinesis was completed (Fig. 1H).

Motile cells were typically 5–8 μm with a single visible flagellum; no eyespot was observed. Swimming cells were spherical to pyriform to oval in shape and had a single parietal chloroplast, 1–3 contractile vacuoles, a chrysolaminarin vacuole but rarely had lipid droplets (Fig. 2, A and B). When stained with Lugol's solution, a second flagellum was not visible (Fig. 2C). Motile cells divided by forming dumbbell shapes and the two daughter cells were separated by a pinching or centripetal constriction type of cytokinesis (Fig. 2D). The plastid typically divided during cytokinesis (Fig. 2D). In some cases, the chloroplast failed to divide, or if divided then failed to segregate, into the two daughter cells, and consequently one of the daughter cells was formed without a chloroplast (Fig. 2, E and F). In some cases, a tiny plastid was observed in one daughter cell but the other daughter cell had a very large plastid (Fig. 2G). For hyponeustonic cells, the two swimming cells separated and rapidly transformed into the typical flagellate cell morphology. Stalked cells occurred when cells attached to around the test tube margin at the air–water interface, and often these cells remained connected by a cytoplasmic strain (Fig. 2, F and G).

Observations using transmission electron microscopy were limited because of the poor fixation quality. Cells had two flagella when viewed with TEM (Fig. 3A). The two flagella were arranged nearly opposite of each other (~180° orientation of the two basal bodies), and the basal bodies overlapped at their proximal ends. The transitional plates of both flagella were dense, and a transitional helix with three gyres was observed (Fig. 3A). The proximal end of the R_1_ root (R_3_ root with Moestrup's 2000 system) was present but the fixation quality did not allow for reconstruction of the flagellar apparatus. Observations using scanning electron microscopy showed one long flagellum bearing mastigonemes (Fig. 3B) and one very short second flagellum (Fig. 3, B and C). Both flagella laid in a shallow depression (Fig. 3C).

*Kremastochrysopsis americana* sp. nov. strain CCMP260 grew hyponeustonically just below the air–water meniscus in recently transferred cultures; bacteria grew at or above the air–water interface (Fig. 4A). In older cultures, many cells sank to the bottom of the test tube, and cells formed cloud‐like masses with cells held together by a thin colonial gel (not shown). The gel margin stained with brilliant cresyl blue but the watery gel matrix did not stain (not shown). Non‐motile vegetative cells were naked (without a cell wall), typically 5–10 μm in diameter, and they contained a single chloroplast, chrysolaminarin vacuole, lipid droplets, and one or two contractile vacuoles (Fig. 4B). When cells were severely flattened by the microscope slide/coverslip, then the chrysolaminarin vacuole became granular, showing a papillose consistency (Fig. 4, C and D); such flattened cells reached diameters of nearly 20 μm before bursting. The chloroplast in smaller cells was parietal and rarely lobed (Fig. 4B); however, in larger cells, the plastids were bilobed (Fig. 4, C and D). When plastids were deeply lobed, a small bridge between the lobes was visible (Fig. 4D). Cell division of immobile cells was like that described for strain DR75b.

Motile cells were typically 5–6 μm wide and 6–10 μm long. They contained a single visible flagellum that was inserted at the anterior end of the cell (Fig. 4, E–G). The chloroplast was parietal and usually trough‐shaped, and it lacked a red eyespot (Fig. 4, E and F). The flagellum was 1–1.5 times as long as the cell and beat with a sinusoidal wave motion. The flagellum appeared longer on cells stained with Lugol's solution, but this was due to shrinkage of the cytoplasm (Fig. 4G). Flagellate cells underwent cell division by producing a second immature flagellum via flagellar transformation (Fig. 4H). The two flagella moved to opposite sides of the mother cell, but the cell remained spheroid in shape (Fig. 4I). The chloroplast divided before there was evidence of cytokinesis (Fig. 4J). Gradually, the mother cell elongated (Fig. 4K), and then cytokinesis occurred quickly to produce two daughter cells. Unlike strain DR75b, CCMP260 rarely produced colorless daughter cells or stalked cells.

No TEM was attempted for *Kremastochrysopsis americana*. SEM observations showed a very short second flagellum that laid in a shallow depression (Fig. 3D).

### Molecular phylogenetic analyses

**Figure 5:**
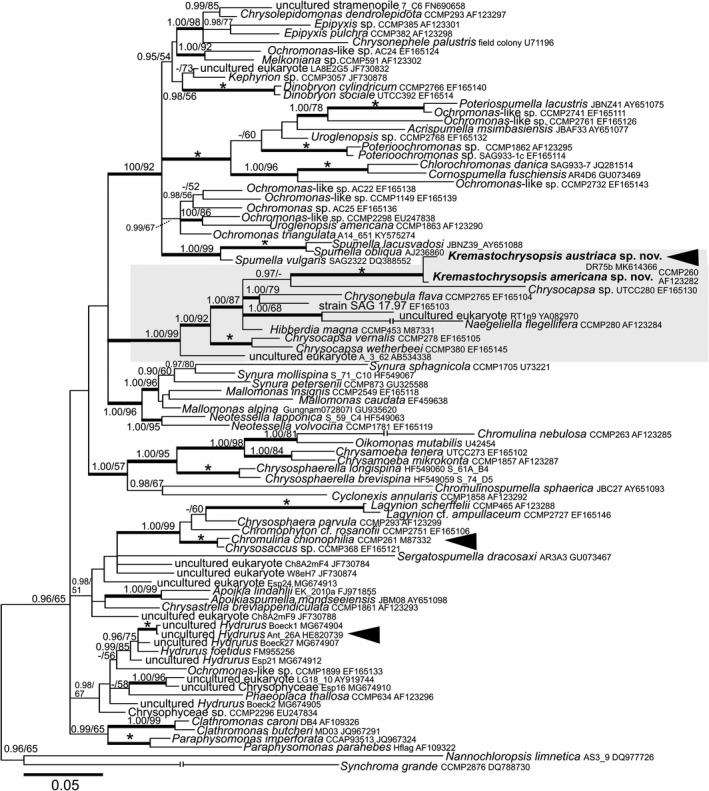
Bayesian phylogenetic tree based on the 18S rRNA gene. The newly described species are in bold. Accession numbers, strain and field sample codes are indicated after each species name. The scale bar shows the estimated number of substitutions per site. Origin in snow is indicated by black arrowheads for relevant species. The Hibberdiales clade is highlighted in a grey box. Posterior probabilities (0.95 or more) and bootstrap values from maximum likelihood analyses (50% or more) are shown. Full statistical support (1.00/100) is marked with an asterisk. Thick branches represent nodes receiving the highest posterior probability support (1.00). For *Naegeliella flagellifera, Chromulina nebulosa*, and *Synchroma grande* branch lengths were shortened by 50% for graphic reasons.

**Figure 6:**
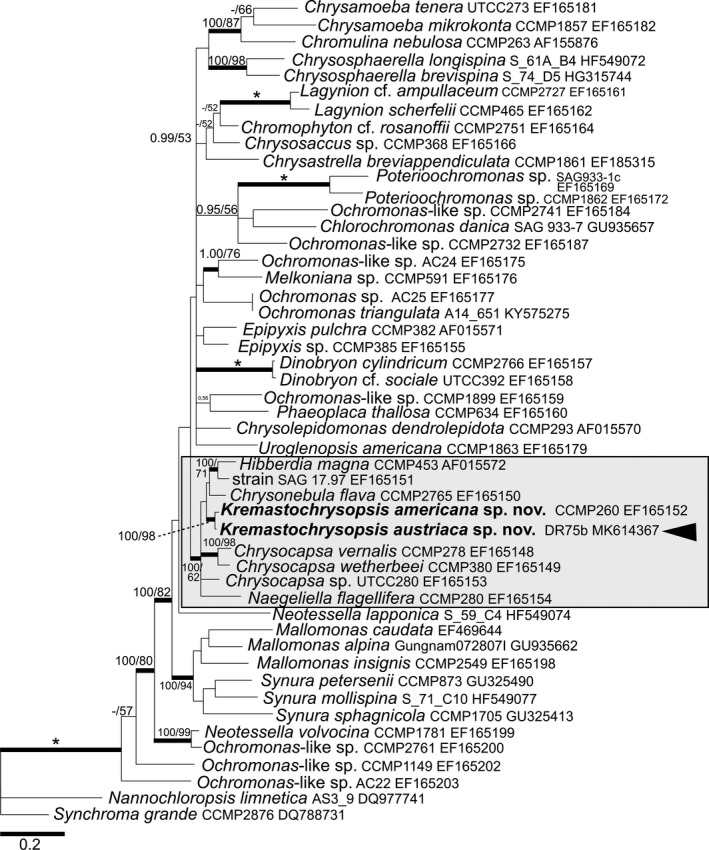
Bayesian phylogenetic tree based on the *rbc*L gene. The newly described species are in bold. Accession numbers, strain, or field sample codes are indicated after each species name. The scale bar shows the estimated number of substitutions per site. Origin in snow is indicated by black arrowhead for relevant species. The Hibberdiales clade is highlighted in a grey box. Posterior probabilities (0.95 or more) and bootstrap values from maximum likelihood analyses (50% or more) are shown. Full statistical support (1.00/100) is marked with an asterisk. Thick branches represent nodes receiving the highest posterior probability support (1.00).

The 18S rRNA gene for the two species differed by 11 nucleotides (out of 1554 bp), and the *rbc*L differed by 14 nucleotides (out of 1035 bp). Based on 18S rRNA gene and *rbc*L phylogenies (Figs. 5, 6), *Kremastochrysopsis austriaca* sp. nov. and *K. americana* sp. nov. represented an independent, well‐supported lineage within the well‐supported order Hibberdiales. These two new species were closely related sister taxa in the 18S rRNA gene tree, and they were in turn sister to strain UTCC280, tentatively identified as *Chrysocapsa* sp. In the *rbc*L tree, *Kremastochrysopsis* occupied a clade with *Hibberdia magna*,* Chrysonebula flava*, and strain SAG 17.97. The phylogenies inferred from the two markers that they were partly incongruent: the *rbc*L phylogenetic tree generally had lower support values for internal branches, resulting in less resolution of taxonomic relationships (Fig. 6). A possible saturation of the *rbc*L data set was checked, the strength of the phylogenetic signal versus noise was assessed for the 18S rRNA gene and different *rbc*L codon partitions (Fig. S1 in the Supporting Information). The significant saturation was revealed for the third *rbc*L codon partition (Fig. S1C). Neither removal of the saturated nucleotide sites by the site‐stripping method nor total deletion of the third *rbc*L codon from alignment improved the reconstructed phylogeny (Figs. S2 and S3 in the Supporting Information). Still, the *rbc*L phylogeny based on the initial *rbc*L alignment was more congruent with the 18S rRNA gene phylogeny than were the two other *rbc*L trees where nucleotide positions were removed.

## Discussion

### Morphology and classification

*Kremastochrysis* is a poorly known genus that was first described from bog waters near Františkovy Lázně, Czech Republic (Pascher 1942). The primary defining character of the genus is its hyponeustonic habit, that is, cells hanging below the water surface. *Kremastochrysis pendens*, the type species, has a single chloroplast. Its vegetative cells are 8–11 μm in diameter, and its zoospores have two flagella visible with a light microscope but no eyespot. *Kremastochrysis minor* was described from a small pond in Castellví de Rosanes in northeastern Spain (Catalan 1987); vegetative cells of *Kremastochrysis minor* are 6–7 μm, with a single parietal chloroplast that occupies the surface farthest from the water surface. Its zoospores are biflagellate, 5 μm in diameter (or elliptical, 5 × 9 μm in size), and each zoospore has a single chloroplast. Both vegetative and zoospore chloroplasts have an eyespot.

Prior to our paper, there was only a single species of *Kremastochrysopsis*,* Kremastochrysopsis ocellata*, which has two chloroplasts per cell; the vegetative cells are up to 25 μm and the zoospores have one LM visible flagellum and a red eyespot on one plastid.

Unfortunately, these *Kremastochrysis* and *Kremastochrysopsis* species have not been reported again. Catalan (1987) designated an ink drawing as the holotype (iconotype) for *Kremastochrysis minor*, but Pascher (1942) did not designate a holotype for either of his two taxa. Therefore, no biological material was used for the nomenclatural type specimens and no DNA can be obtained from the iconotype. Ideally, recollection of Pascher's two taxa from the type locality and gene sequencing would be the best approach because one could establish DNA sequences for the type species (e.g., see Andersen et al. 2017). Such a search for the type species from the type locality is beyond the scope of this study, and therefore we identified our strains as *Kremastochrysopsis* based upon their light microscopic morphology. For nomenclatural purposes, we designated an ink drawing as the lectotype specimen for each of Pascher's taxa.

The cells of *Chrysotilos ferrea* resemble those of *Kremastochrysopsis. Chrysotilos ferrea* is also a neustonic organism and it was found in very shallow pools formed by melted snow at mountain meadows near Lunz, Austria (Pascher 1931). Cells are 7–9 μm in size with a single parietal chloroplast; it produces dorsoventrally flattened uniflagellate zoospores (as viewed by light microscopy) with an eyespot. The cells form yellow‐brown to black‐brown flakes (up to 1 mm) on the water surface, but it is unclear if these are epineustonic or hyponeustonic layers. A thin gelatinous envelope surrounds the cells of a flake. A second species, *Chrysotilos tatrica*, was described from the epineuston of a small pools near the cable car station Gubałówka, Zakopane (Krakow region), Poland (Czosnowski 1948). The vegetative cells are spherical, 7–9 μm in diameter, with a single golden chloroplast with a small eyespot. Uniflagellate zoospores are 7–10 μm by 5.5–7 μm. Thus, *Chrysotilos* vegetative and swimming cells are similar to *Kremastochrysopsis*, but there are two important differences that separate the genera. First, *Chrysotilos* produces pseudocysts‐shell‐like structures that become heavily impregnated with iron (Pascher 1931, Czosnowski 1948). The pseudocysts typically have two parts, upper and lower “halves.” Furthermore, *Chrysotilos* produces sporangia‐like structures that contain 2–32 or more cells. While *Kremastochrysopsis* may produce palmelloid colonies in old cultures, the cells are evenly dispersed and they are never enclosed to produce a sporangium‐like structure.

Finally, because *Kremastochrysopsis austriaca* was collected in melting snow rather than the neuston, we compare our strains to the uniflagellate snow alga, *Chromulina chionophilia*. *Chromulina chionophila* has flattened cells, one flagellum (as viewed in the light microscope), and an eyespot in the chloroplast (Stein 1963). *Chromulina chionophilia* differs from our new species because of the flattened cells and the eyespot. There is some resemblance between the swimming cells of *Chrysotilos* and *Chromulina chionophilia,* but the latter does not form pseudocysts or sporangia.

### Molecular phylogeny

Our phylogenies (18S rRNA and *rbc*L genes) show that both of our new *Kremastochrysopsis* species are members of monophyletic Hibberdiales clade. *Kremastochrysopsis austriaca* showed no close phylogenetic relation to other chrysophytes causing blooms in melting snow (Figs. 5, 6), for example, *Chromulina chionophilia* (Hoham 1975), *Hydrurus* sp. (Remias et al. 2013), or *Ochromonas‐*like flagellates (Tanabe et al. 2011).

### Ecology

The occurrence of *Kremastochrysopsis austriaca* in melting mountain snow causing a yellowish bloom was surprising because other members of the Hibberdiales are known from standing or flowing waters. Previous studies found that *Kremastochrysopsis austriaca* occurred together with other “snow algae genera,” for example, *Chloromonas* and *Sanguina* (Remias et al. 2018, Procházková et al. 2019). While additional studies must be made, it seems probable that *K. austriaca* is distributed in other areas of the Austrian Alps.

DR acknowledges funding from the Austrian Science Fund (FWF): P29959. LP and LN acknowledge funding from the Czech Science Foundation (GACR): 18‐02634S. We are grateful to Pavel Škaloud (Charles University, Prague, Czech Republic) for consultations about applying the modified site‐stripping approach. We express our thanks to Ivan Čepička and Miroslav Hyliš (both: Charles University, Prague, Czech Republic) for their recommendations concerning chemical fixation for scanning electron microscopy and the flagellar apparatus observation.

## Supporting information


**Figure S1.** Plots of DNA codon substitutional saturation. Maximum likelihood‐corrected distances are plotted against uncorrected p‐distances for the first (A), second (B) and third (C) codon position of the *rbc*L gene, and (D) the 18S rRNA gene dataset. Strong curving of saturation plots indicates the significant saturation of molecular datasets. The lowest corrected distance used for removal of fast‐evolving sites is indicated by an arrow.

**Figure S2.** Bayesian phylogenetic tree of Chrysophyta based on the partitioned *rbc*L dataset after removal of saturated sites by the site‐stripping method. The newly described species are in bold. Origin in snow is indicated for relevant species. The Hibberdiales clade is highlighted in a grey box. Posterior probabilities (0.95 or more) and bootstrap values from maximum likelihood analyses (50% or more) are shown. Full statistical support (1.00/100) is marked with an asterisk. Thick branches represent nodes receiving the highest posterior probability support (1.00). Accession numbers, strain or field sample codes are indicated after each species name. The scale bar shows the estimated number of substitutions per site.

**Figure S3.** Bayesian phylogenetic tree of Chrysophyta based on the partitioned *rbc*L dataset after removal of the third codon positions. The newly described species are in bold. Origin in snow is indicated for relevant species. The Hibberdiales clade is highlighted in a grey box. Posterior probabilities (0.95 or more) and bootstrap values from maximum likelihood analyses (50% or more) are shown. Full statistical support (1.00/100) is marked with an asterisk. Thick branches represent nodes receiving the highest posterior probability support (1.00). Accession numbers, strain or field sample codes are indicated after each species name. The scale bar shows the estimated number of substitutions per site.
