# Supplementary figures and images for: Two New Kremastochrysopsis species, K. austriaca sp. nov. and K. americana sp. nov. (Chrysophyceae)1
Source: J Phycol. 2019 Nov 27;56(1):135–45. doi: 10.1111/jpy.12937 (PMC7054049; doi:10.1111/jpy.12937)

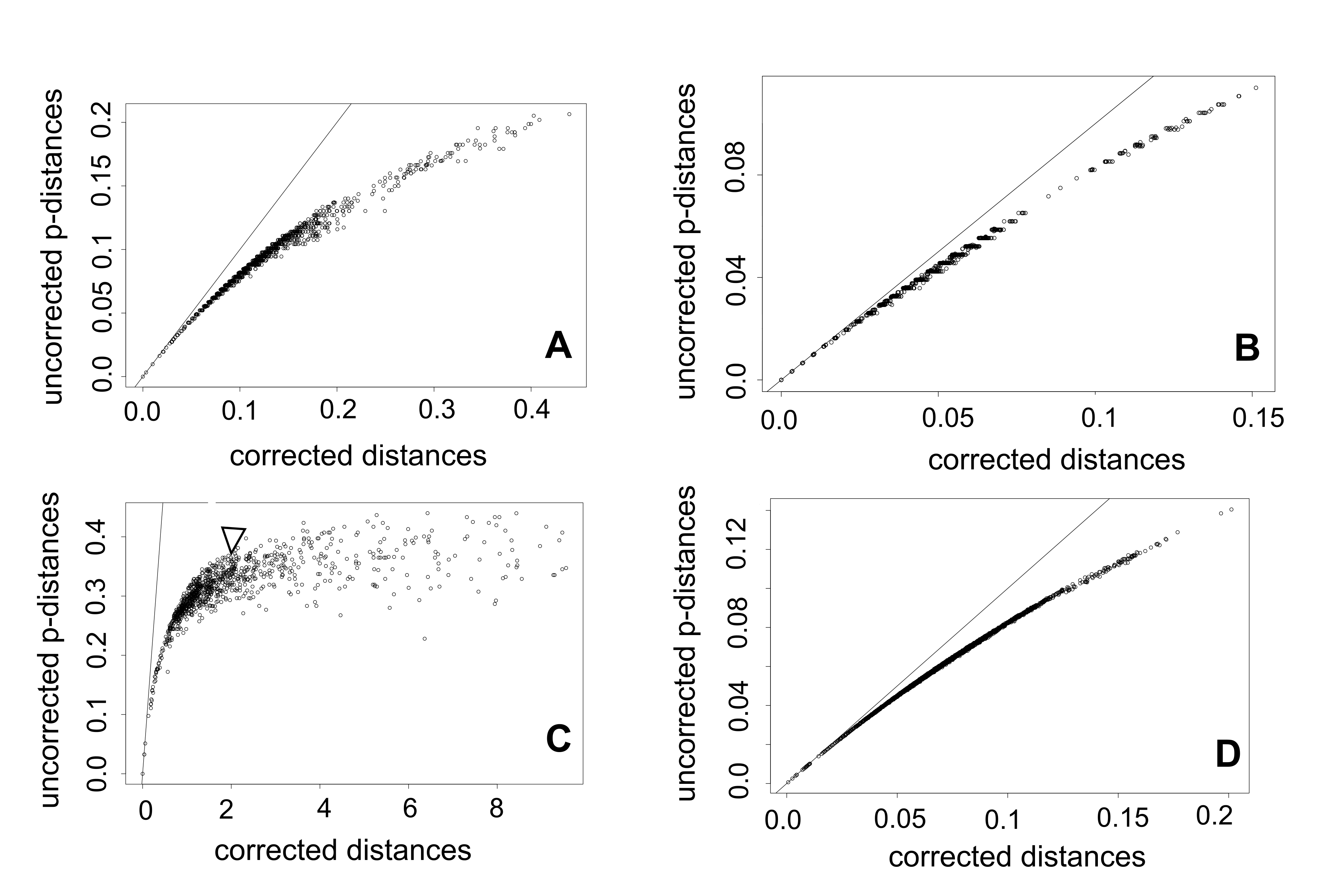

Supplement: Supplementary file 1 — Figure S1. Plots of DNA codon substitutional saturation. Maximum likelihood‐corrected distances are plotted against uncorrected p‐distances for the first (A), second (B) and third (C) codon position of the rbcL gene, and (D) the 18S rRNA gene dataset. Strong curving of saturation plots indicates the significant saturation of molecular datasets. The lowest corrected distance used for removal of fast‐evolving sites is indicated by an arrow. [file JPY-56-135-s001.tif]

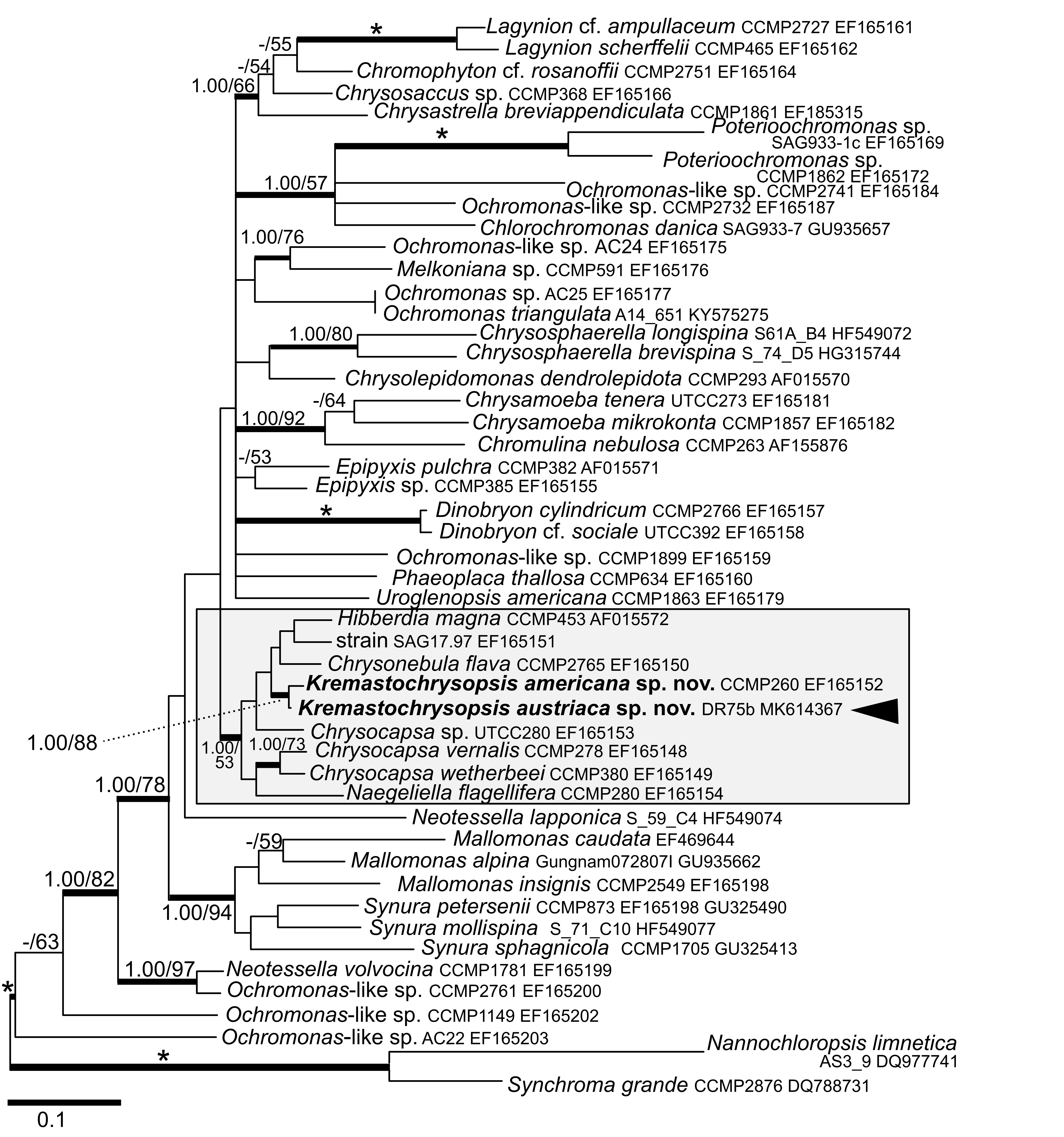

Supplement: Supplementary file 2 — Figure S2. Bayesian phylogenetic tree of Chrysophyta based on the partitioned rbcL dataset after removal of saturated sites by the site‐stripping method. The newly described species are in bold. Origin in snow is indicated for relevant species. The Hibberdiales clade is highlighted in a grey box. Posterior probabilities (0.95 or more) and bootstrap values from maximum likelihood analyses (50% or more) are shown. Full statistical support (1.00/100) is marked with an asterisk. Thick branches represent nodes receiving the highest posterior probability support (1.00). Accession numbers, strain or field sample codes are indicated after each species name. The scale bar shows the estimated number of substitutions per site. [file JPY-56-135-s002.tif]

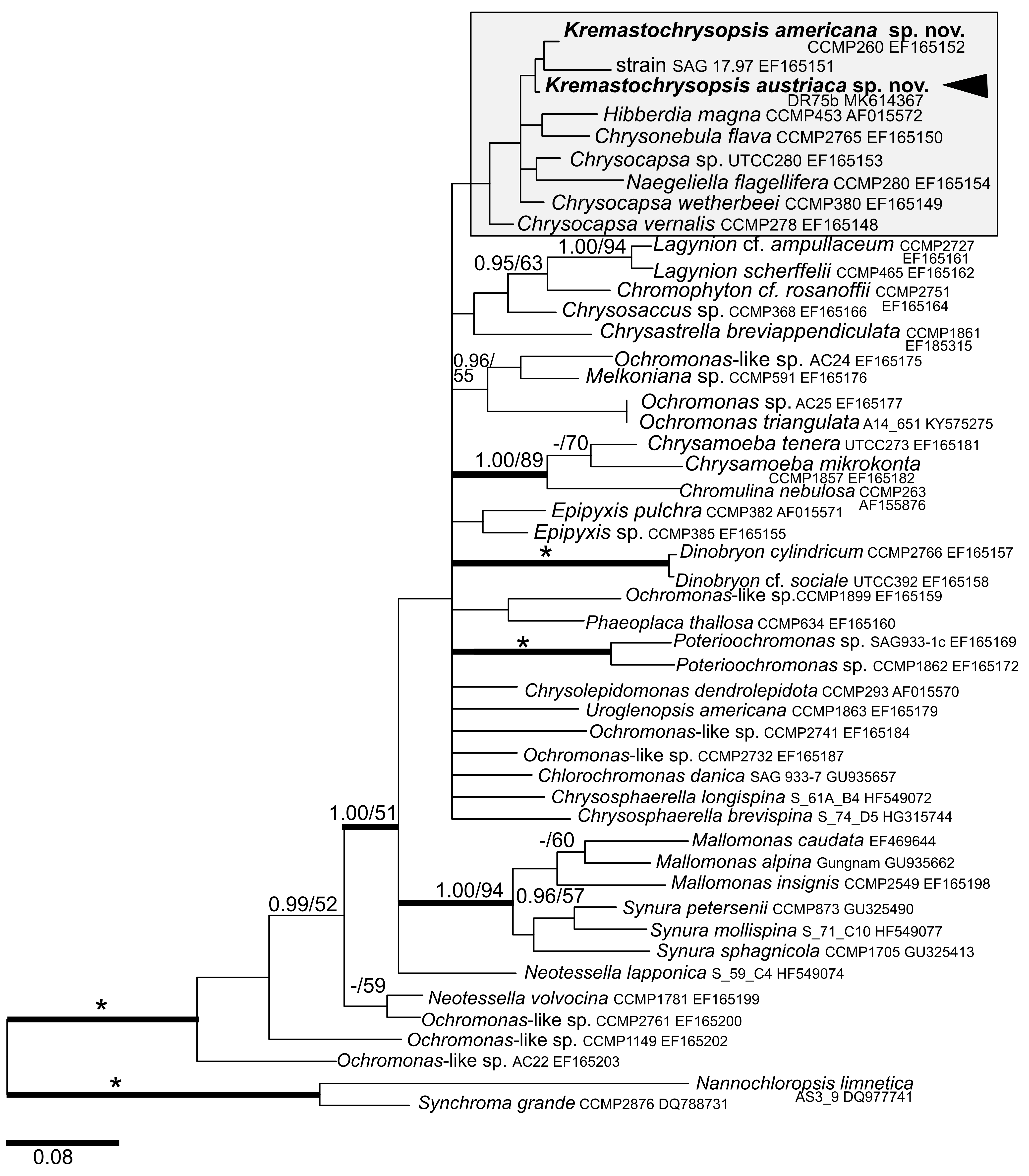

Supplement: Supplementary file 3 — Figure S3. Bayesian phylogenetic tree of Chrysophyta based on the partitioned rbcL dataset after removal of the third codon positions. The newly described species are in bold. Origin in snow is indicated for relevant species. The Hibberdiales clade is highlighted in a grey box. Posterior probabilities (0.95 or more) and bootstrap values from maximum likelihood analyses (50% or more) are shown. Full statistical support (1.00/100) is marked with an asterisk. Thick branches represent nodes receiving the highest posterior probability support (1.00). Accession numbers, strain or field sample codes are indicated after each species name. The scale bar shows the estimated number of substitutions per site. [file JPY-56-135-s003.tif]
